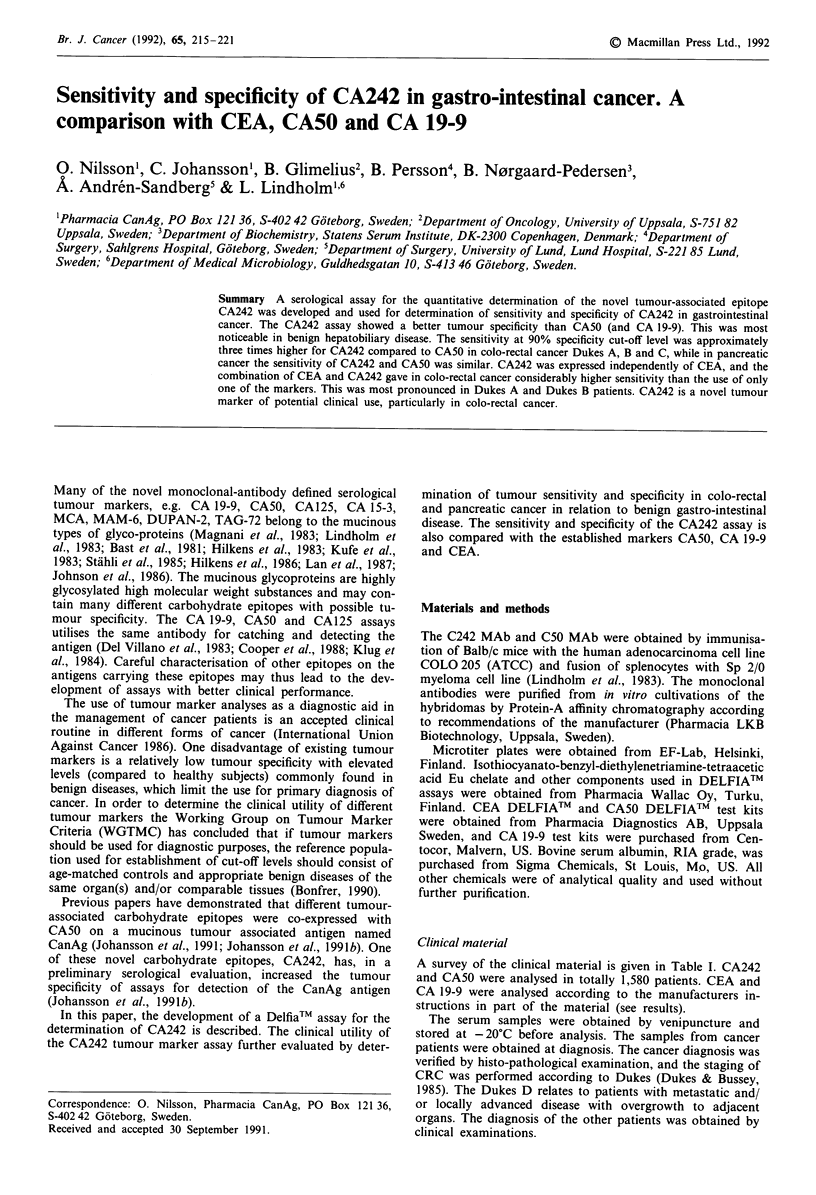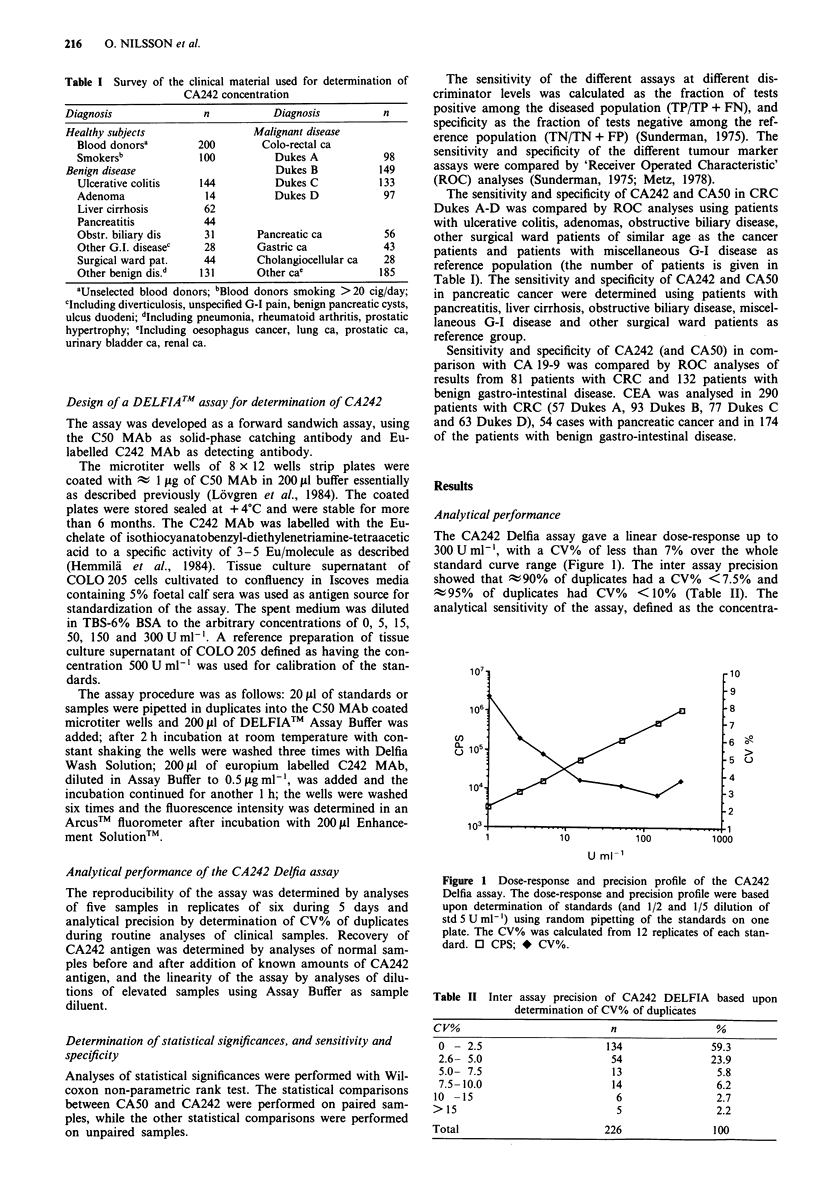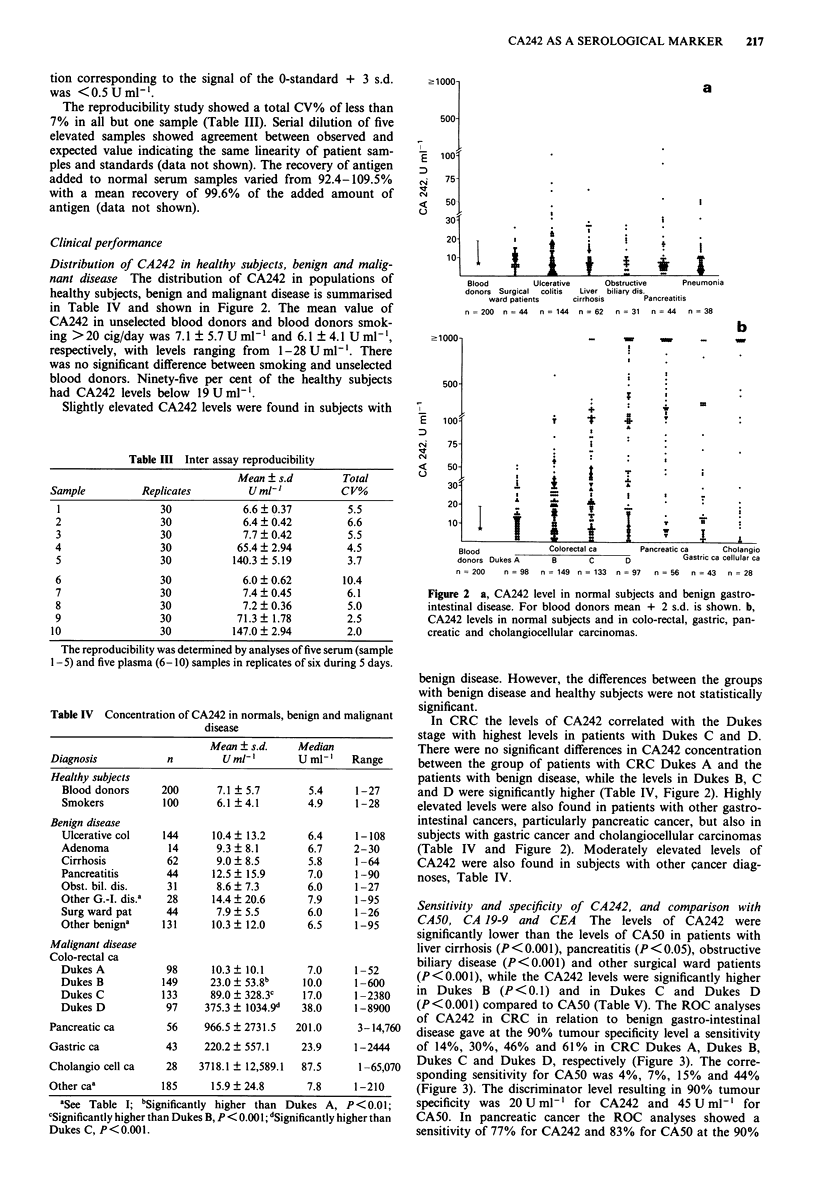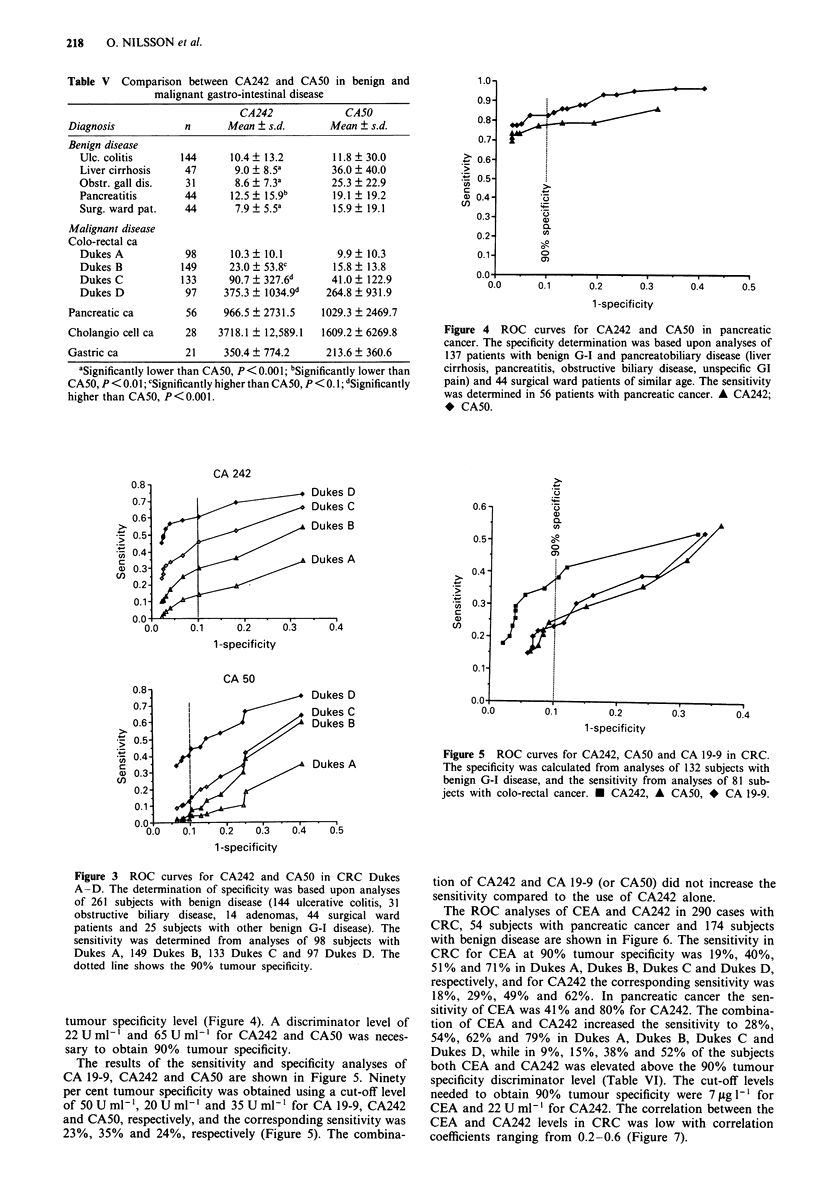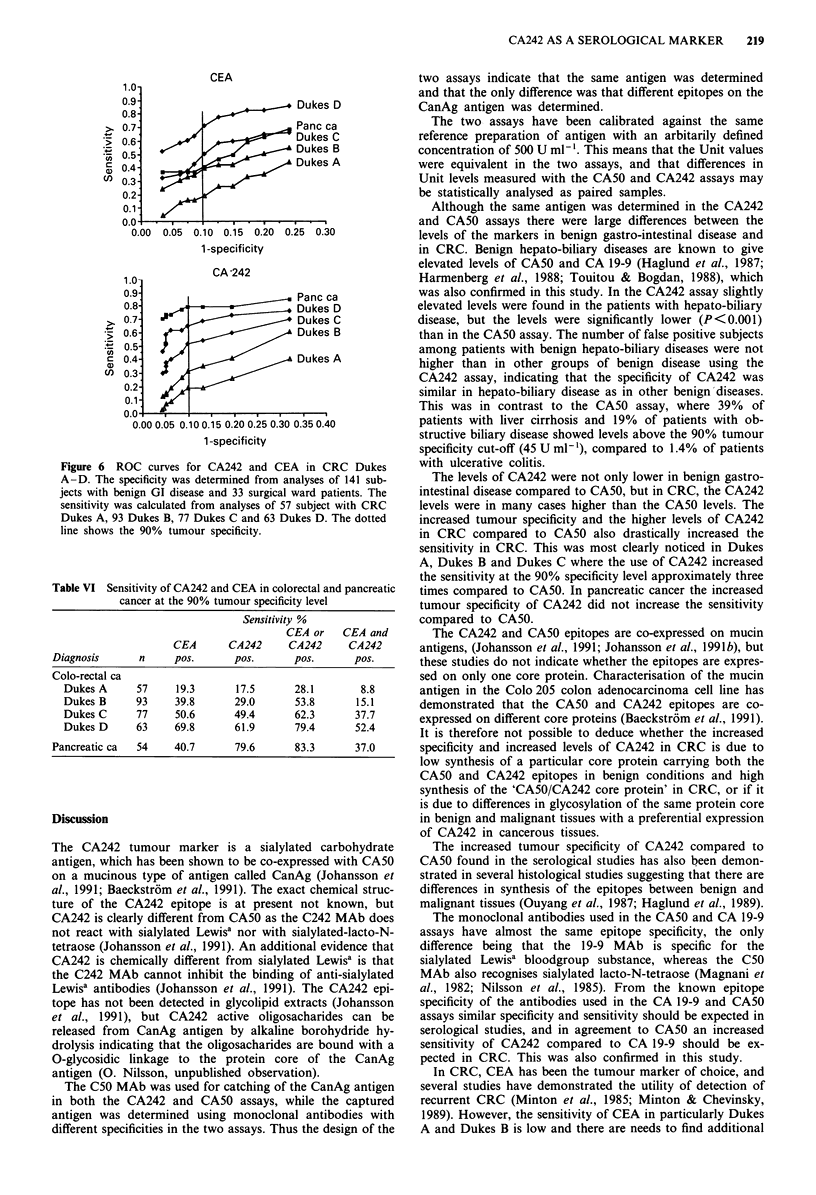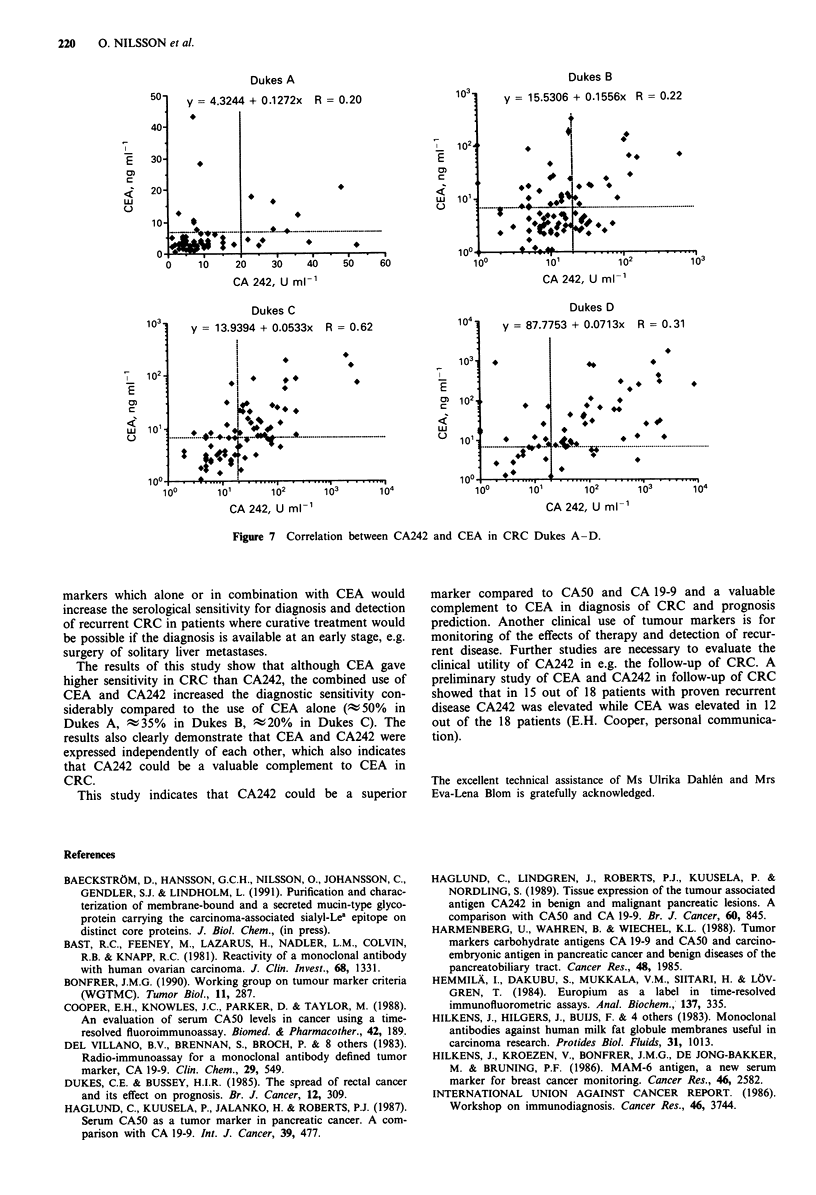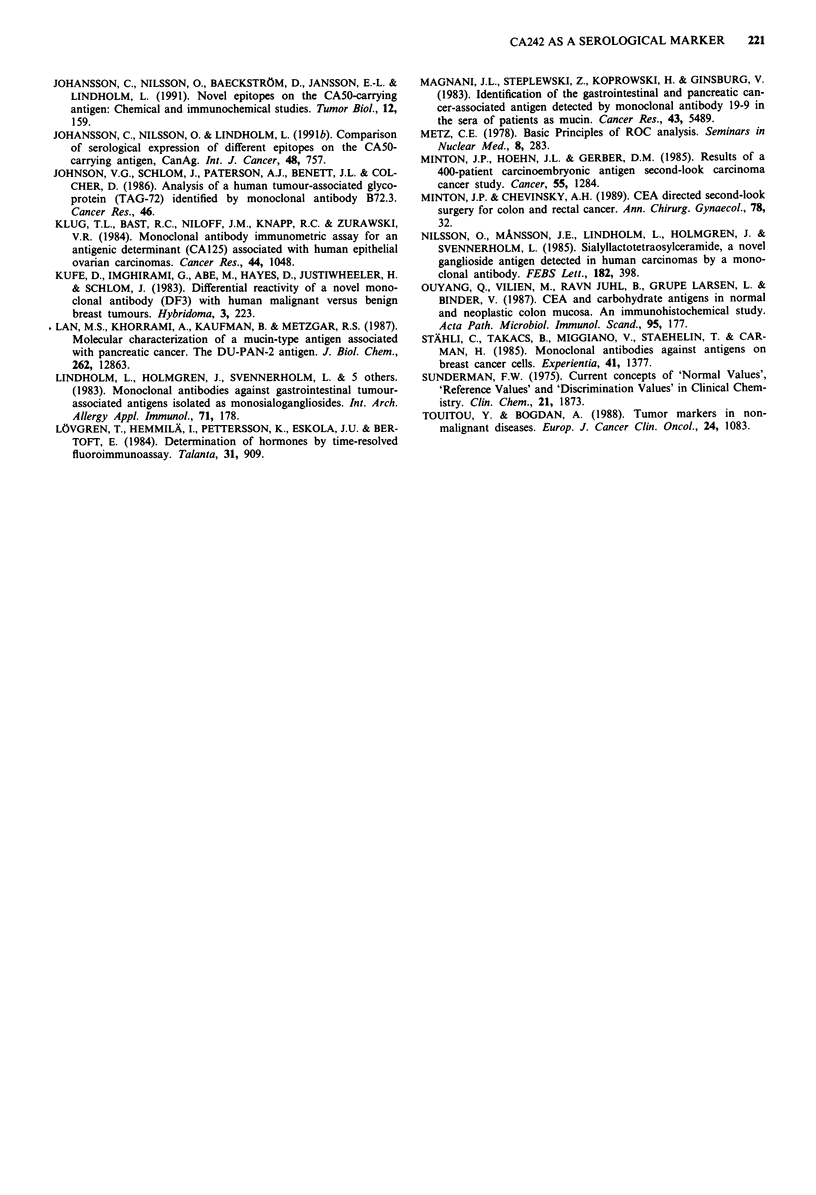# Sensitivity and specificity of CA242 in gastro-intestinal cancer. A comparison with CEA, CA50 and CA 19-9.

**DOI:** 10.1038/bjc.1992.44

**Published:** 1992-02

**Authors:** O. Nilsson, C. Johansson, B. Glimelius, B. Persson, B. Nørgaard-Pedersen, A. Andrén-Sandberg, L. Lindholm

**Affiliations:** Pharmacia CanAg, Göteborg, Sweden.

## Abstract

A serological assay for the quantitative determination of the novel tumour-associated epitope CA242 was developed and used for determination of sensitivity and specificity of CA242 in gastrointestinal cancer. The CA242 assay showed a better tumour specificity than CA50 (and CA 19-9). This was most noticeable in benign hepatobiliary disease. The sensitivity at 90% specificity cut-off level was approximately three times higher for CA242 compared to CA50 in colo-rectal cancer Dukes A, B and C, while in pancreatic cancer the sensitivity of CA242 and CA50 was similar. CA242 was expressed independently of CEA, and the combination of CEA and CA242 gave in colo-rectal cancer considerably higher sensitivity than the use of only one of the markers. This was most pronounced in Dukes A and Dukes B patients. CA242 is a novel tumour marker of potential clinical use, particularly in colo-rectal cancer.


Br. J. Cancer (1992), 65, 215-221 © Macmillan Press Ltd., 1992

### Sensitivity and specificity of CA242 in gastro-intestinal cancer. A comparison with CEA, CA50 and CA 19-9

O. Nilsson[1], C. Johansson[1], B. Glimelius[2], B. Persson[4], B. Nørgaard-Pedersen[3], Å. Andrén-Sandberg[5] & L. Lindholm[1,6]

[1]*Pharmacia CanAg, PO Box 121 36, S-402 42 Göteborg, Sweden;* [2]*Department of Oncology, University of Uppsala, S-751 82 Uppsala, Sweden;* [3]*Department of Biochemistry, Statens Serum Institute, DK-2300 Copenhagen, Denmark;* [4]*Department of Surgery, Sahlgrens Hospital, Göteborg, Sweden;* [5]*Department of Surgery, University of Lund, Lund Hospital, S-221 85 Lund, Sweden;* [6]*Department of Medical Microbiology, Guldhedsgatan 10, S-413 46 Göteborg, Sweden.*

**Summary** A serological assay for the quantitative determination of the novel tumour-associated epitope CA242 was developed and used for determination of sensitivity and specificity of CA242 in gastrointestinal cancer. The CA242 assay showed a better tumour specificity than CA50 (and CA 19-9). This was most noticeable in benign hepatobiliary disease. The sensitivity at 90% specificity cut-off level was approximately three times higher for CA242 compared to CA50 in colo-rectal cancer Dukes A, B and C, while in pancreatic cancer the sensitivity of CA242 and CA50 was similar. CA242 was expressed independently of CEA, and the combination of CEA and CA242 gave in colo-rectal cancer considerably higher sensitivity than the use of only one of the markers. This was most pronounced in Dukes A and Dukes B patients. CA242 is a novel tumour marker of potential clinical use, particularly in colo-rectal cancer.

Many of the novel monoclonal-antibody defined serological tumour markers, e.g. CA 19-9, CA50, CA125, CA 15-3, MCA, MAM-6, DUPAN-2, TAG-72 belong to the mucinous types of glyco-proteins (Magnani *et al.*, 1983; Lindholm *et al.*, 1983; Bast *et al.*, 1981; Hilkens *et al.*, 1983; Kufe *et al.*, 1983; Stähli *et al.*, 1985; Hilkens *et al.*, 1986; Lan *et al.*, 1987; Johnson *et al.*, 1986). The mucinous glycoproteins are highly glycosylated high molecular weight substances and may contain many different carbohydrate epitopes with possible tumour specificity. The CA 19-9, CA50 and CA125 assays utilises the same antibody for catching and detecting the antigen (Del Villano *et al.*, 1983; Cooper *et al.*, 1988; Klug *et al.*, 1984). Careful characterisation of other epitopes on the antigens carrying these epitopes may thus lead to the development of assays with better clinical performance.

The use of tumour marker analyses as a diagnostic aid in the management of cancer patients is an accepted clinical routine in different forms of cancer (International Union Against Cancer 1986). One disadvantage of existing tumour markers is a relatively low tumour specificity with elevated levels (compared to healthy subjects) commonly found in benign diseases, which limit the use for primary diagnosis of cancer. In order to determine the clinical utility of different tumour markers the Working Group on Tumour Marker Criteria (WGTMC) has concluded that if tumour markers should be used for diagnostic purposes, the reference population used for establishment of cut-off levels should consist of age-matched controls and appropriate benign diseases of the same organ(s) and/or comparable tissues (Bonfrer, 1990).

Previous papers have demonstrated that different tumour-associated carbohydrate epitopes were co-expressed with CA50 on a mucinous tumour associated antigen named CanAg (Johansson *et al.*, 1991; Johansson *et al.*, 1991*b*). One of these novel carbohydrate epitopes, CA242, has, in a preliminary serological evaluation, increased the tumour specificity of assays for detection of the CanAg antigen (Johansson *et al.*, 1991*b*).

In this paper, the development of a Delfia™ assay for the determination of CA242 is described. The clinical utility of the CA242 tumour marker assay further evaluated by determination of tumour sensitivity and specificity in colo-rectal and pancreatic cancer in relation to benign gastro-intestinal disease. The sensitivity and specificity of the CA242 assay is also compared with the established markers CA50, CA 19-9 and CEA.

Correspondence: O. Nilsson, Pharmacia CanAg, PO Box 121 36, S-402 42 Göteborg, Sweden.
Received and accepted 30 September 1991.

### Materials and methods

The C242 MAb and C50 MAb were obtained by immunisation of Balb/c mice with the human adenocarcinoma cell line COLO 205 (ATCC) and fusion of splenocytes with Sp 2/0 myeloma cell line (Lindholm *et al.*, 1983). The monoclonal antibodies were purified from *in vitro* cultivations of the hybridomas by Protein-A affinity chromatography according to recommendations of the manufacturer (Pharmacia LKB Biotechnology, Uppsala, Sweden).

Microtiter plates were obtained from EF-Lab, Helsinki, Finland. Isothiocyanato-benzyl-diethylenetriamine-tetraacetic acid Eu chelate and other components used in DELFIA™ assays were obtained from Pharmacia Wallac Oy, Turku, Finland. CEA DELFIA™ and CA50 DELFIA™ test kits were obtained from Pharmacia Diagnostics AB, Uppsala Sweden, and CA 19-9 test kits were purchased from Centocor, Malvern, US. Bovine serum albumin, RIA grade, was purchased from Sigma Chemicals, St Louis, Mo, US. All other chemicals were of analytical quality and used without further purification.

### *Clinical material*

A survey of the clinical material is given in Table I. CA242 and CA50 were analysed in totally 1,580 patients. CEA and CA 19-9 were analysed according to the manufacturers instructions in part of the material (see results).

The serum samples were obtained by venipuncture and stored at $-20°C$ before analysis. The samples from cancer patients were obtained at diagnosis. The cancer diagnosis was verified by histo-pathological examination, and the staging of CRC was performed according to Dukes (Dukes & Bussey, 1985). The Dukes D relates to patients with metastatic and/or locally advanced disease with overgrowth to adjacent organs. The diagnosis of the other patients was obtained by clinical examinations.

**Table I** Survey of the clinical material used for determination of CA242 concentration

| *Diagnosis* | *n* | *Diagnosis* | *n* |
|---|---|---|---|
| *Healthy subjects* | | *Malignant disease* | |
| Blood donors[a] | 200 | Colo-rectal ca | |
| Smokers[b] | 100 | Dukes A | 98 |
| *Benign disease* | | Dukes B | 149 |
| Ulcerative colitis | 144 | Dukes C | 133 |
| Adenoma | 14 | Dukes D | 97 |
| Liver cirrhosis | 62 | | |
| Pancreatitis | 44 | | |
| Obstr. biliary dis | 31 | Pancreatic ca | 56 |
| Other G.I. disease[c] | 28 | Gastric ca | 43 |
| Surgical ward pat. | 44 | Cholangiocellular ca | 28 |
| Other benign dis.[d] | 131 | Other ca[e] | 185 |

[a]Unselected blood donors; [b]Blood donors smoking >20 cig/day; [c]Including diverticulosis, unspecified G-I pain, benign pancreatic cysts, ulcus duodeni; [d]Including pneumonia, rheumatoid arthritis, prostatic hypertrophy; [e]Including oesophagus cancer, lung ca, prostatic ca, urinary bladder ca, renal ca.

### *Design of a DELFIA™ assay for determination of CA242*

The assay was developed as a forward sandwich assay, using the C50 MAb as solid-phase catching antibody and Eu-labelled C242 MAb as detecting antibody.

The microtiter wells of 8 × 12 wells strip plates were coated with ≈ 1 µg of C50 MAb in 200 µl buffer essentially as described previously (Lövgren *et al.*, 1984). The coated plates were stored sealed at +4°C and were stable for more than 6 months. The C242 MAb was labelled with the Eu-chelate of isothiocyanatobenzyl-diethylenetriamine-tetraacetic acid to a specific activity of 3–5 Eu/molecule as described (Hemmilä *et al.*, 1984). Tissue culture supernatant of COLO 205 cells cultivated to confluency in Iscoves media containing 5% foetal calf sera was used as antigen source for standardization of the assay. The spent medium was diluted in TBS-6% BSA to the arbitrary concentrations of 0, 5, 15, 50, 150 and 300 U ml$^{-1}$. A reference preparation of tissue culture supernatant of COLO 205 defined as having the concentration 500 U ml$^{-1}$ was used for calibration of the standards.

The assay procedure was as follows: 20 µl of standards or samples were pipetted in duplicates into the C50 MAb coated microtiter wells and 200 µl of DELFIA™ Assay Buffer was added; after 2 h incubation at room temperature with constant shaking the wells were washed three times with Delfia Wash Solution; 200 µl of europium labelled C242 MAb, diluted in Assay Buffer to 0.5 µg ml$^{-1}$, was added and the incubation continued for another 1 h; the wells were washed six times and the fluorescence intensity was determined in an Arcus™ fluorometer after incubation with 200 µl Enhancement Solution™.

### *Analytical performance of the CA242 Delfia assay*

The reproducibility of the assay was determined by analyses of five samples in replicates of six during 5 days and analytical precision by determination of CV% of duplicates during routine analyses of clinical samples. Recovery of CA242 antigen was determined by analyses of normal samples before and after addition of known amounts of CA242 antigen, and the linearity of the assay by analyses of dilutions of elevated samples using Assay Buffer as sample diluent.

### *Determination of statistical significances, and sensitivity and specificity*

Analyses of statistical significances were performed with Wilcoxon non-parametric rank test. The statistical comparisons between CA50 and CA242 were performed on paired samples, while the other statistical comparisons were performed on unpaired samples.

The sensitivity of the different assays at different discriminator levels was calculated as the fraction of tests positive among the diseased population (TP/TP + FN), and specificity as the fraction of tests negative among the reference population (TN/TN + FP) (Sunderman, 1975). The sensitivity and specificity of the different tumour marker assays were compared by 'Receiver Operated Characteristic' (ROC) analyses (Sunderman, 1975; Metz, 1978).

The sensitivity and specificity of CA242 and CA50 in CRC Dukes A-D was compared by ROC analyses using patients with ulcerative colitis, adenomas, obstructive biliary disease, other surgical ward patients of similar age as the cancer patients and patients with miscellaneous G-I disease as reference population (the number of patients is given in Table I). The sensitivity and specificity of CA242 and CA50 in pancreatic cancer were determined using patients with pancreatitis, liver cirrhosis, obstructive biliary disease, miscellaneous G-I disease and other surgical ward patients as reference group.

Sensitivity and specificity of CA242 (and CA50) in comparison with CA 19-9 was compared by ROC analyses of results from 81 patients with CRC and 132 patients with benign gastro-intestinal disease. CEA was analysed in 290 patients with CRC (57 Dukes A, 93 Dukes B, 77 Dukes C and 63 Dukes D), 54 cases with pancreatic cancer and in 174 of the patients with benign gastro-intestinal disease.

### Results

### *Analytical performance*

The CA242 Delfia assay gave a linear dose-response up to 300 U ml$^{-1}$, with a CV% of less than 7% over the whole standard curve range (Figure 1). The inter assay precision showed that ≈90% of duplicates had a CV% <7.5% and ≈95% of duplicates had CV% <10% (Table II). The analytical sensitivity of the assay, defined as the concentra-

**Figure 1** Dose-response and precision profile of the CA242 Delfia assay. The dose-response and precision profile were based upon determination of standards (and 1/2 and 1/5 dilution of std 5 U ml$^{-1}$) using random pipetting of the standards on one plate. The CV% was calculated from 12 replicates of each standard. □ CPS; ◆ CV%.

**Table II** Inter assay precision of CA242 DELFIA based upon determination of CV% of duplicates

| *CV%* | *n* | *%* |
|---|---|---|
| 0 – 2.5 | 134 | 59.3 |
| 2.6– 5.0 | 54 | 23.9 |
| 5.0– 7.5 | 13 | 5.8 |
| 7.5–10.0 | 14 | 6.2 |
| 10 –15 | 6 | 2.7 |
| >15 | 5 | 2.2 |
| Total | 226 | 100 |

tion corresponding to the signal of the 0-standard + 3 s.d. was $<0.5\ U\ ml^{-1}$.

The reproducibility study showed a total CV% of less than 7% in all but one sample (Table III). Serial dilution of five elevated samples showed agreement between observed and expected value indicating the same linearity of patient samples and standards (data not shown). The recovery of antigen added to normal serum samples varied from 92.4–109.5% with a mean recovery of 99.6% of the added amount of antigen (data not shown).

### *Clinical performance*

*Distribution of CA242 in healthy subjects, benign and malignant disease* The distribution of CA242 in populations of healthy subjects, benign and malignant disease is summarised in Table IV and shown in Figure 2. The mean value of CA242 in unselected blood donors and blood donors smoking >20 cig/day was $7.1 \pm 5.7\ U\ ml^{-1}$ and $6.1 \pm 4.1\ U\ ml^{-1}$, respectively, with levels ranging from $1-28\ U\ ml^{-1}$. There was no significant difference between smoking and unselected blood donors. Ninety-five per cent of the healthy subjects had CA242 levels below $19\ U\ ml^{-1}$.

Slightly elevated CA242 levels were found in subjects with benign disease. However, the differences between the groups with benign disease and healthy subjects were not statistically significant.

**Table III** Inter assay reproducibility

| Sample | Replicates | *Mean ± s.d* $U\ ml^{-1}$ | *Total CV%* |
|---|---|---|---|
| 1 | 30 | 6.6 ± 0.37 | 5.5 |
| 2 | 30 | 6.4 ± 0.42 | 6.6 |
| 3 | 30 | 7.7 ± 0.42 | 5.5 |
| 4 | 30 | 65.4 ± 2.94 | 4.5 |
| 5 | 30 | 140.3 ± 5.19 | 3.7 |
| 6 | 30 | 6.0 ± 0.62 | 10.4 |
| 7 | 30 | 7.4 ± 0.45 | 6.1 |
| 8 | 30 | 7.2 ± 0.36 | 5.0 |
| 9 | 30 | 71.3 ± 1.78 | 2.5 |
| 10 | 30 | 147.0 ± 2.94 | 2.0 |

The reproducibility was determined by analyses of five serum (sample 1–5) and five plasma (6–10) samples in replicates of six during 5 days.

**Table IV** Concentration of CA242 in normals, benign and malignant disease

| *Diagnosis* | *n* | *Mean ± s.d.* $U\ ml^{-1}$ | *Median* $U\ ml^{-1}$ | Range |
|---|---|---|---|---|
| *Healthy subjects* | | | | |
| Blood donors | 200 | 7.1 ± 5.7 | 5.4 | 1–27 |
| Smokers | 100 | 6.1 ± 4.1 | 4.9 | 1–28 |
| *Benign disease* | | | | |
| Ulcerative col | 144 | 10.4 ± 13.2 | 6.4 | 1–108 |
| Adenoma | 14 | 9.3 ± 8.1 | 6.7 | 2–30 |
| Cirrhosis | 62 | 9.0 ± 8.5 | 5.8 | 1–64 |
| Pancreatitis | 44 | 12.5 ± 15.9 | 7.0 | 1–90 |
| Obst. bil. dis. | 31 | 8.6 ± 7.3 | 6.0 | 1–27 |
| Other G.-I. dis.[a] | 28 | 14.4 ± 20.6 | 7.9 | 1–95 |
| Surg ward pat | 44 | 7.9 ± 5.5 | 6.0 | 1–26 |
| Other benign[a] | 131 | 10.3 ± 12.0 | 6.5 | 1–95 |
| *Malignant disease* | | | | |
| Colo-rectal ca | | | | |
| Dukes A | 98 | 10.3 ± 10.1 | 7.0 | 1–52 |
| Dukes B | 149 | 23.0 ± 53.8[b] | 10.0 | 1–600 |
| Dukes C | 133 | 89.0 ± 328.3[c] | 17.0 | 1–2380 |
| Dukes D | 97 | 375.3 ± 1034.9[d] | 38.0 | 1–8900 |
| Pancreatic ca | 56 | 966.5 ± 2731.5 | 201.0 | 3–14,760 |
| Gastric ca | 43 | 220.2 ± 557.1 | 23.9 | 1–2444 |
| Cholangio cell ca | 28 | 3718.1 ± 12,589.1 | 87.5 | 1–65,070 |
| Other ca[a] | 185 | 15.9 ± 24.8 | 7.8 | 1–210 |

[a]See Table I; [b]Significantly higher than Dukes A, $P<0.01$; [c]Significantly higher than Dukes B, $P<0.001$; [d]Significantly higher than Dukes C, $P<0.001$.

**Figure 2** **a**, CA242 level in normal subjects and benign gastro-intestinal disease. For blood donors mean + 2 s.d. is shown. **b**, CA242 levels in normal subjects and in colo-rectal, gastric, pancreatic and cholangiocellular carcinomas.

In CRC the levels of CA242 correlated with the Dukes stage with highest levels in patients with Dukes C and D. There were no significant differences in CA242 concentration between the group of patients with CRC Dukes A and the patients with benign disease, while the levels in Dukes B, C and D were significantly higher (Table IV, Figure 2). Highly elevated levels were also found in patients with other gastro-intestinal cancers, particularly pancreatic cancer, but also in subjects with gastric cancer and cholangiocellular carcinomas (Table IV and Figure 2). Moderately elevated levels of CA242 were also found in subjects with other cancer diagnoses, Table IV.

*Sensitivity and specificity of CA242, and comparison with CA50, CA 19-9 and CEA* The levels of CA242 were significantly lower than the levels of CA50 in patients with liver cirrhosis ($P<0.001$), pancreatitis ($P<0.05$), obstructive biliary disease ($P<0.001$) and other surgical ward patients ($P<0.001$), while the CA242 levels were significantly higher in Dukes B ($P<0.1$) and in Dukes C and Dukes D ($P<0.001$) compared to CA50 (Table V). The ROC analyses of CA242 in CRC in relation to benign gastro-intestinal disease gave at the 90% tumour specificity level a sensitivity of 14%, 30%, 46% and 61% in CRC Dukes A, Dukes B, Dukes C and Dukes D, respectively (Figure 3). The corresponding sensitivity for CA50 was 4%, 7%, 15% and 44% (Figure 3). The discriminator level resulting in 90% tumour specificity was $20\ U\ ml^{-1}$ for CA242 and $45\ U\ ml^{-1}$ for CA50. In pancreatic cancer the ROC analyses showed a sensitivity of 77% for CA242 and 83% for CA50 at the 90%

**Table V** Comparison between CA242 and CA50 in benign and malignant gastro-intestinal disease

| *Diagnosis* | *n* | *CA242* Mean ± s.d. | *CA50* Mean ± s.d. |
|---|---|---|---|
| *Benign disease* | | | |
| Ulc. colitis | 144 | 10.4 ± 13.2 | 11.8 ± 30.0 |
| Liver cirrhosis | 47 | 9.0 ± 8.5[a] | 36.0 ± 40.0 |
| Obstr. gall dis. | 31 | 8.6 ± 7.3[a] | 25.3 ± 22.9 |
| Pancreatitis | 44 | 12.5 ± 15.9[b] | 19.1 ± 19.2 |
| Surg. ward pat. | 44 | 7.9 ± 5.5[a] | 15.9 ± 19.1 |
| *Malignant disease* | | | |
| Colo-rectal ca | | | |
| Dukes A | 98 | 10.3 ± 10.1 | 9.9 ± 10.3 |
| Dukes B | 149 | 23.0 ± 53.8[c] | 15.8 ± 13.8 |
| Dukes C | 133 | 90.7 ± 327.6[d] | 41.0 ± 122.9 |
| Dukes D | 97 | 375.3 ± 1034.9[d] | 264.8 ± 931.9 |
| Pancreatic ca | 56 | 966.5 ± 2731.5 | 1029.3 ± 2469.7 |
| Cholangio cell ca | 28 | 3718.1 ± 12,589.1 | 1609.2 ± 6269.8 |
| Gastric ca | 21 | 350.4 ± 774.2 | 213.6 ± 360.6 |

[a]Significantly lower than CA50, $P<0.001$; [b]Significantly lower than CA50, $P<0.01$; [c]Significantly higher than CA50, $P<0.1$; [d]Significantly higher than CA50, $P<0.001$.

**Figure 4** ROC curves for CA242 and CA50 in pancreatic cancer. The specificity determination was based upon analyses of 137 patients with benign G-I and pancreatobiliary disease (liver cirrhosis, pancreatitis, obstructive biliary disease, unspecific GI pain) and 44 surgical ward patients of similar age. The sensitivity was determined in 56 patients with pancreatic cancer. ▲ CA242; ◆ CA50.

**Figure 3** ROC curves for CA242 and CA50 in CRC Dukes A–D. The determination of specificity was based upon analyses of 261 subjects with benign disease (144 ulcerative colitis, 31 obstructive biliary disease, 14 adenomas, 44 surgical ward patients and 25 subjects with other benign G-I disease). The sensitivity was determined from analyses of 98 subjects with Dukes A, 149 Dukes B, 133 Dukes C and 97 Dukes D. The dotted line shows the 90% tumour specificity.

**Figure 5** ROC curves for CA242, CA50 and CA 19-9 in CRC. The specificity was calculated from analyses of 132 subjects with benign G-I disease, and the sensitivity from analyses of 81 subjects with colo-rectal cancer. ■ CA242, ▲ CA50, ◆ CA 19-9.

tumour specificity level (Figure 4). A discriminator level of 22 U ml$^{-1}$ and 65 U ml$^{-1}$ for CA242 and CA50 was necessary to obtain 90% tumour specificity.

The results of the sensitivity and specificity analyses of CA 19-9, CA242 and CA50 are shown in Figure 5. Ninety per cent tumour specificity was obtained using a cut-off level of 50 U ml$^{-1}$, 20 U ml$^{-1}$ and 35 U ml$^{-1}$ for CA 19-9, CA242 and CA50, respectively, and the corresponding sensitivity was 23%, 35% and 24%, respectively (Figure 5). The combination of CA242 and CA 19-9 (or CA50) did not increase the sensitivity compared to the use of CA242 alone.

The ROC analyses of CEA and CA242 in 290 cases with CRC, 54 subjects with pancreatic cancer and 174 subjects with benign disease are shown in Figure 6. The sensitivity in CRC for CEA at 90% tumour specificity was 19%, 40%, 51% and 71% in Dukes A, Dukes B, Dukes C and Dukes D, respectively, and for CA242 the corresponding sensitivity was 18%, 29%, 49% and 62%. In pancreatic cancer the sensitivity of CEA was 41% and 80% for CA242. The combination of CEA and CA242 increased the sensitivity to 28%, 54%, 62% and 79% in Dukes A, Dukes B, Dukes C and Dukes D, while in 9%, 15%, 38% and 52% of the subjects both CEA and CA242 was elevated above the 90% tumour specificity discriminator level (Table VI). The cut-off levels needed to obtain 90% tumour specificity were 7 μg l$^{-1}$ for CEA and 22 U ml$^{-1}$ for CA242. The correlation between the CEA and CA242 levels in CRC was low with correlation coefficients ranging from 0.2–0.6 (Figure 7).

**Figure 6** ROC curves for CA242 and CEA in CRC Dukes A–D. The specificity was determined from analyses of 141 subjects with benign GI disease and 33 surgical ward patients. The sensitivity was calculated from analyses of 57 subject with CRC Dukes A, 93 Dukes B, 77 Dukes C and 63 Dukes D. The dotted line shows the 90% tumour specificity.

**Table VI** Sensitivity of CA242 and CEA in colorectal and pancreatic cancer at the 90% tumour specificity level

| | | Sensitivity % | | | |
|---|---|---|---|---|---|
| *Diagnosis* | *n* | *CEA pos.* | *CA242 pos.* | *CEA or CA242 pos.* | *CEA and CA242 pos.* |
| Colo-rectal ca | | | | | |
| Dukes A | 57 | 19.3 | 17.5 | 28.1 | 8.8 |
| Dukes B | 93 | 39.8 | 29.0 | 53.8 | 15.1 |
| Dukes C | 77 | 50.6 | 49.4 | 62.3 | 37.7 |
| Dukes D | 63 | 69.8 | 61.9 | 79.4 | 52.4 |
| Pancreatic ca | 54 | 40.7 | 79.6 | 83.3 | 37.0 |

### Discussion

The CA242 tumour marker is a sialylated carbohydrate antigen, which has been shown to be co-expressed with CA50 on a mucinous type of antigen called CanAg (Johansson *et al.*, 1991; Baeckström *et al.*, 1991). The exact chemical structure of the CA242 epitope is at present not known, but CA242 is clearly different from CA50 as the C242 MAb does not react with sialylated Lewis[a] nor with sialylated-lacto-N-tetraose (Johansson *et al.*, 1991). An additional evidence that CA242 is chemically different from sialylated Lewis[a] is that the C242 MAb cannot inhibit the binding of anti-sialylated Lewis[a] antibodies (Johansson *et al.*, 1991). The CA242 epitope has not been detected in glycolipid extracts (Johansson *et al.*, 1991), but CA242 active oligosacharides can be released from CanAg antigen by alkaline borohydride hydrolysis indicating that the oligosacharides are bound with a O-glycosidic linkage to the protein core of the CanAg antigen (O. Nilsson, unpublished observation).

The C50 MAb was used for catching of the CanAg antigen in both the CA242 and CA50 assays, while the captured antigen was determined using monoclonal antibodies with different specificities in the two assays. Thus the design of the two assays indicate that the same antigen was determined and that the only difference was that different epitopes on the CanAg antigen was determined.

The two assays have been calibrated against the same reference preparation of antigen with an arbitarily defined concentration of 500 U $ml^{-1}$. This means that the Unit values were equivalent in the two assays, and that differences in Unit levels measured with the CA50 and CA242 assays may be statistically analysed as paired samples.

Although the same antigen was determined in the CA242 and CA50 assays there were large differences between the levels of the markers in benign gastro-intestinal disease and in CRC. Benign hepato-biliary diseases are known to give elevated levels of CA50 and CA 19-9 (Haglund *et al.*, 1987; Harmenberg *et al.*, 1988; Touitou & Bogdan, 1988), which was also confirmed in this study. In the CA242 assay slightly elevated levels were found in the patients with hepato-biliary disease, but the levels were significantly lower ($P<0.001$) than in the CA50 assay. The number of false positive subjects among patients with benign hepato-biliary diseases were not higher than in other groups of benign disease using the CA242 assay, indicating that the specificity of CA242 was similar in hepato-biliary disease as in other benign diseases. This was in contrast to the CA50 assay, where 39% of patients with liver cirrhosis and 19% of patients with obstructive biliary disease showed levels above the 90% tumour specificity cut-off (45 U $ml^{-1}$), compared to 1.4% of patients with ulcerative colitis.

The levels of CA242 were not only lower in benign gastro-intestinal disease compared to CA50, but in CRC, the CA242 levels were in many cases higher than the CA50 levels. The increased tumour specificity and the higher levels of CA242 in CRC compared to CA50 also drastically increased the sensitivity in CRC. This was most clearly noticed in Dukes A, Dukes B and Dukes C where the use of CA242 increased the sensitivity at the 90% specificity level approximately three times compared to CA50. In pancreatic cancer the increased tumour specificity of CA242 did not increase the sensitivity compared to CA50.

The CA242 and CA50 epitopes are co-expressed on mucin antigens, (Johansson *et al.*, 1991; Johansson *et al.*, 1991*b*), but these studies do not indicate whether the epitopes are expressed on only one core protein. Characterisation of the mucin antigen in the Colo 205 colon adenocarcinoma cell line has demonstrated that the CA50 and CA242 epitopes are co-expressed on different core proteins (Baeckström *et al.*, 1991). It is therefore not possible to deduce whether the increased specificity and increased levels of CA242 in CRC is due to low synthesis of a particular core protein carrying both the CA50 and CA242 epitopes in benign conditions and high synthesis of the 'CA50/CA242 core protein' in CRC, or if it is due to differences in glycosylation of the same protein core in benign and malignant tissues with a preferential expression of CA242 in cancerous tissues.

The increased tumour specificity of CA242 compared to CA50 found in the serological studies has also been demonstrated in several histological studies suggesting that there are differences in synthesis of the epitopes between benign and malignant tissues (Ouyang *et al.*, 1987; Haglund *et al.*, 1989).

The monoclonal antibodies used in the CA50 and CA 19-9 assays have almost the same epitope specificity, the only difference being that the 19-9 MAb is specific for the sialylated Lewis[a] bloodgroup substance, whereas the C50 MAb also recognises sialylated lacto-N-tetraose (Magnani *et al.*, 1982; Nilsson *et al.*, 1985). From the known epitope specificity of the antibodies used in the CA 19-9 and CA50 assays similar specificity and sensitivity should be expected in serological studies, and in agreement to CA50 an increased sensitivity of CA242 compared to CA 19-9 should be expected in CRC. This was also confirmed in this study.

In CRC, CEA has been the tumour marker of choice, and several studies have demonstrated the utility of detection of recurrent CRC (Minton *et al.*, 1985; Minton & Chevinsky, 1989). However, the sensitivity of CEA in particularly Dukes A and Dukes B is low and there are needs to find additional

**Figure 7** Correlation between CA242 and CEA in CRC Dukes A–D.

markers which alone or in combination with CEA would increase the serological sensitivity for diagnosis and detection of recurrent CRC in patients where curative treatment would be possible if the diagnosis is available at an early stage, e.g. surgery of solitary liver metastases.

The results of this study show that although CEA gave higher sensitivity in CRC than CA242, the combined use of CEA and CA242 increased the diagnostic sensitivity considerably compared to the use of CEA alone ($\approx$50% in Dukes A, $\approx$35% in Dukes B, $\approx$20% in Dukes C). The results also clearly demonstrate that CEA and CA242 were expressed independently of each other, which also indicates that CA242 could be a valuable complement to CEA in CRC.

This study indicates that CA242 could be a superior marker compared to CA50 and CA 19-9 and a valuable complement to CEA in diagnosis of CRC and prognosis prediction. Another clinical use of tumour markers is for monitoring of the effects of therapy and detection of recurrent disease. Further studies are necessary to evaluate the clinical utility of CA242 in e.g. the follow-up of CRC. A preliminary study of CEA and CA242 in follow-up of CRC showed that in 15 out of 18 patients with proven recurrent disease CA242 was elevated while CEA was elevated in 12 out of the 18 patients (E.H. Cooper, personal communication).

The excellent technical assistance of Ms Ulrika Dahlén and Mrs Eva-Lena Blom is gratefully acknowledged.

### References

BAECKSTRÖM, D., HANSSON, G.C.H., NILSSON, O., JOHANSSON, C., GENDLER, S.J. & LINDHOLM, L. (1991). Purification and characterization of membrane-bound and a secreted mucin-type glycoprotein carrying the carcinoma-associated sialyl-Le$^a$ epitope on distinct core proteins. *J. Biol. Chem.*, (in press).

BAST, R.C., FEENEY, M., LAZARUS, H., NADLER, L.M., COLVIN, R.B. & KNAPP, R.C. (1981). Reactivity of a monoclonal antibody with human ovarian carcinoma. *J. Clin. Invest.*, **68**, 1331.

BONFRER, J.M.G. (1990). Working group on tumour marker criteria (WGTMC). *Tumor Biol.*, **11**, 287.

COOPER, E.H., KNOWLES, J.C., PARKER, D. & TAYLOR, M. (1988). An evaluation of serum CA50 levels in cancer using a time-resolved fluoroimmunoassay. *Biomed. & Pharmacother.*, **42**, 189.

DEL VILLANO, B.V., BRENNAN, S., BROCH, P. & 8 others (1983). Radio-immunoassay for a monoclonal antibody defined tumor marker, CA 19-9. *Clin. Chem.*, **29**, 549.

DUKES, C.E. & BUSSEY, H.I.R. (1985). The spread of rectal cancer and its effect on prognosis. *Br. J. Cancer*, **12**, 309.

HAGLUND, C., KUUSELA, P., JALANKO, H. & ROBERTS, P.J. (1987). Serum CA50 as a tumor marker in pancreatic cancer. A comparison with CA 19-9. *Int. J. Cancer*, **39**, 477.

HAGLUND, C., LINDGREN, J., ROBERTS, P.J., KUUSELA, P. & NORDLING, S. (1989). Tissue expression of the tumour associated antigen CA242 in benign and malignant pancreatic lesions. A comparison with CA50 and CA 19-9. *Br. J. Cancer*, **60**, 845.

HARMENBERG, U., WAHREN, B. & WIECHEL, K.L. (1988). Tumor markers carbohydrate antigens CA 19-9 and CA50 and carcinoembryonic antigen in pancreatic cancer and benign diseases of the pancreatobiliary tract. *Cancer Res.*, **48**, 1985.

HEMMILÄ, I., DAKUBU, S., MUKKALA, V.M., SIITARI, H. & LÖVGREN, T. (1984). Europium as a label in time-resolved immunofluorometric assays. *Anal. Biochem.*, **137**, 335.

HILKENS, J., HILGERS, J., BUIJS, F. & 4 others (1983). Monoclonal antibodies against human milk fat globule membranes useful in carcinoma research. *Protides Biol. Fluids*, **31**, 1013.

HILKENS, J., KROEZEN, V., BONFRER, J.M.G., DE JONG-BAKKER, M. & BRUNING, P.F. (1986). MAM-6 antigen, a new serum marker for breast cancer monitoring. *Cancer Res.*, **46**, 2582.

INTERNATIONAL UNION AGAINST CANCER REPORT. (1986). Workshop on immunodiagnosis. *Cancer Res.*, **46**, 3744.

JOHANSSON, C., NILSSON, O., BAECKSTRÖM, D., JANSSON, E.-L. & LINDHOLM, L. (1991). Novel epitopes on the CA50-carrying antigen: Chemical and immunochemical studies. *Tumor Biol.*, **12**, 159.

JOHANSSON, C., NILSSON, O. & LINDHOLM, L. (1991*b*). Comparison of serological expression of different epitopes on the CA50-carrying antigen, CanAg. *Int. J. Cancer*, **48**, 757.

JOHNSON, V.G., SCHLOM, J., PATERSON, A.J., BENETT, J.L. & COLCHER, D. (1986). Analysis of a human tumour-associated glycoprotein (TAG-72) identified by monoclonal antibody B72.3. *Cancer Res.*, **46**.

KLUG, T.L., BAST, R.C., NILOFF, J.M., KNAPP, R.C. & ZURAWSKI, V.R. (1984). Monoclonal antibody immunometric assay for an antigenic determinant (CA125) associated with human epithelial ovarian carcinomas. *Cancer Res.*, **44**, 1048.

KUFE, D., IMGHIRAMI, G., ABE, M., HAYES, D., JUSTIWHEELER, H. & SCHLOM, J. (1983). Differential reactivity of a novel monoclonal antibody (DF3) with human malignant versus benign breast tumours. *Hybridoma*, **3**, 223.

LAN, M.S., KHORRAMI, A., KAUFMAN, B. & METZGAR, R.S. (1987). Molecular characterization of a mucin-type antigen associated with pancreatic cancer. The DU-PAN-2 antigen. *J. Biol. Chem.*, **262**, 12863.

LINDHOLM, L., HOLMGREN, J., SVENNERHOLM, L. & 5 others. (1983). Monoclonal antibodies against gastrointestinal tumour-associated antigens isolated as monosialogangliosides. *Int. Arch. Allergy Appl. Immunol.*, **71**, 178.

LÖVGREN, T., HEMMILÄ, I., PETTERSSON, K., ESKOLA, J.U. & BERTOFT, E. (1984). Determination of hormones by time-resolved fluoroimmunoassay. *Talanta*, **31**, 909.

MAGNANI, J.L., STEPLEWSKI, Z., KOPROWSKI, H. & GINSBURG, V. (1983). Identification of the gastrointestinal and pancreatic cancer-associated antigen detected by monoclonal antibody 19-9 in the sera of patients as mucin. *Cancer Res.*, **43**, 5489.

METZ, C.E. (1978). Basic Principles of ROC analysis. *Seminars in Nuclear Med.*, **8**, 283.

MINTON, J.P., HOEHN, J.L. & GERBER, D.M. (1985). Results of a 400-patient carcinoembryonic antigen second-look carcinoma cancer study. *Cancer*, **55**, 1284.

MINTON, J.P. & CHEVINSKY, A.H. (1989). CEA directed second-look surgery for colon and rectal cancer. *Ann. Chirurg. Gynaecol.*, **78**, 32.

NILSSON, O., MÅNSSON, J.E., LINDHOLM, L., HOLMGREN, J. & SVENNERHOLM, L. (1985). Sialyllactotetraosylceramide, a novel ganglioside antigen detected in human carcinomas by a monoclonal antibody. *FEBS Lett.*, **182**, 398.

OUYANG, Q., VILIEN, M., RAVN JUHL, B., GRUPE LARSEN, L. & BINDER, V. (1987). CEA and carbohydrate antigens in normal and neoplastic colon mucosa. An immunohistochemical study. *Acta Path. Microbiol. Immunol. Scand.*, **95**, 177.

STÄHLI, C., TAKACS, B., MIGGIANO, V., STAEHELIN, T. & CARMAN, H. (1985). Monoclonal antibodies against antigens on breast cancer cells. *Experientia*, **41**, 1377.

SUNDERMAN, F.W. (1975). Current concepts of 'Normal Values', 'Reference Values' and 'Discrimination Values' in Clinical Chemistry. *Clin. Chem.*, **21**, 1873.

TOUITOU, Y. & BOGDAN, A. (1988). Tumor markers in non-malignant diseases. *Europ. J. Cancer Clin. Oncol.*, **24**, 1083.